# BPP Bioportide™-mediated (genetic) transformation in cyanobacteria: a rapid and simplified approach for efficient molecular translocation and genome modification

**DOI:** 10.3389/fpls.2026.1812316

**Published:** 2026-04-22

**Authors:** Soumila Mondal, Janette T. Alford, Christoph Kutzner, Karl Forchhammer, Sofia Doello

**Affiliations:** 1 Department of Microbiology/Organismic Interactions, Interfaculty Institute for Microbiology and Infection Medicine, University of Tübingen, Tübingen, Germany; 2 Department of Biology, Washington University, St. Louis, MO, United States; 3 Badische Peptide & Proteine GmbH, Mannheim, Germany; 4 Department of Microbiology, Institute for Integrative Biology of the Cell, University of Paris-Saclay, Gif-sur-Yvette, France

**Keywords:** cyanobacteria, genetic engineering, transformation, Synechocystis, *Synechococcus*, UTEX 3153, PCC 11901

## Abstract

Cyanobacteria are promising candidates for sustainable bioproduction due to their ability to fix CO_2_ via photosynthesis to produce valuable compounds. However, efforts toward their genetic engineering remain limited by inefficient, strain-specific transformation methods. In this study, we systematically evaluated BPP Bioportides™, a protein-based DNA delivery system, as a novel transformation approach across multiple cyanobacterial strains, including *Synechocystis* sp. PCC 6803, *Synechococcus elongatus* PCC 7942, and *Synechococcus* sp. UTEX 3153. BPP Bioportide™ variants BP-17 and BP-12 significantly improved transformation efficiency of both plasmid and linear DNA, even with minimal DNA input of 10 ng, surpassing conventional methods and enabling modification of previously non-model strains. Optimization revealed a narrow window for DNA uptake and identified key factors influencing genomic integration, such as DNA type, host ploidy, and selection conditions. Successful double homologous recombination and partial to full genomic segregation were validated by colony PCR. BPP Bioportide™-mediated transformation offers a versatile and efficient platform for cyanobacterial genome engineering, supporting advances in synthetic biology and carbon-neutral biotechnology.

## 1 Introduction

Cyanobacteria, often referred to as blue-green algae, are among the oldest and most versatile microorganisms on Earth (Jassim et al., 2023). These Gram-negative, prokaryotic, photoautotrophic bacteria have played a crucial role in shaping Earth’s atmosphere by contributing significantly to the Great Oxygenation Event over 2.4 billion years ago (Cavalier-Smith, 2006). As primary producers, cyanobacteria play a significant role in ecological and biogeochemical cycles, forming the foundation of many aquatic ecosystems. In addition, many cyanobacteria fix atmospheric nitrogen and thrive in diverse environments, making them essential players in maintaining global biodiversity and ecosystem stability (Nawaz et al., 2024).

Over the past few decades, research on cyanobacteria has expanded drastically, especially in the area of genetic engineering. Scientists have sought to harness the potential of cyanobacteria as sustainable wholecell factories to produce biofuels, bioplastics, pharmaceuticals, and even carbon sequestration technologies (Savakis and Hellingwerf, 2015). The ability to implement genetic modifications in cyanobacteria offers promising solutions to address global challenges, including climate change, food security, and sustainable energy production. These genetic modifications involve introducing foreign DNA to alter their metabolic pathways or inducing new metabolic pathways to enhance their resilience or optimize the yield of target products. This has opened up new avenues in synthetic biology, biotechnology, and environmental engineering (Berla et al., 2013).

Currently, transformation of cyanobacteria is achieved via various established techniques, including CRISPR-Cas, electroporation, conjugation, and natural transformation (Klähn et al., 2024). These methods facilitate the integration of foreign genes into a cyanobacterial background, enabling enhanced growth rates, increased production of diverse target metabolites, or the generation of strains for environmental remediation. However, longstanding methods based on natural transformation are often inefficient, timeconsuming, and require high DNA concentrations of up to 20 µg/ml for optimal transformation (Zang et al., 2007). While innovative methods like CRISPR-based genome editing reduce time and increase efficiency, they also generate new problems, such as off-target effects (Cheng et al., 2023). Additionally, some cyanobacteria exhibit restriction-modification (R-M) systems and endogenous nucleases, which degrade exogenous DNA and further reduce transformation efficiency, particularly in non-model or environmental isolates (Lyra et al., 2000). The slow growth rates of many cyanobacterial strains under photoautotrophic conditions also limit the throughput of genetic manipulation and screening (Cui et al., 2023). Furthermore, as many cyanobacterial species are polyploid (Griese et al., 2011), multiple gene copies must be replaced to obtain stable and fully segregated mutants, often requiring prolonged selection regimes (Pope et al., 2020). Thus, despite significant progress, challenges such as low transformation efficiency and incomplete segregation remain. These limitations necessitate continuous innovation and refinement of genetic engineering approaches to further establish cyanobacteria as a biotechnological chassis.

Here, we establish a novel genetic modification method for cyanobacteria using transmembrane proteins known as BPP Bioportides™ (BPs), a newly created patent-protected designer protein (Badische Peptide und Proteine GmbH, Mannheim, Germany), providing a powerful tool to explore further the potential of cyanobacteria for sustainable biotechnological advancements and environmental applications.

## 2 Materials and methods

### 2.1 Growth conditions

*Synechocystis* sp. PCC 6803 (glucose-tolerant) wild type (WT) and *Synechococcus elongatus* PCC 7942 WT were routinely maintained in BG11 medium (Rippka, 1992) under continuous 80 µmol Photosynthetically Active Radiation (PAR, 300–700 nm) light and 130 rpm shaking. *Synechococcus* sp.UTEX 3153 WT was routinely maintained in AD7 medium (Stevens et al., 1973) supplemented with vitamin B12 under continuous 80 µmol PAR (300–700 nm) light and 130 rpm shaking (Włodarczyk et al., 2020).

### 2.2 Generation of linear PCR product for transformation of *Synechocystis* sp. PCC 6803

To test the transformation of *Synechocystis* sp. PCC 6803 with linear DNA, the *pirC* knockout cassette was amplified via PCR from the plasmid pJA1 (Orthwein et al., 2025) with primers 3 and 4 (**Table 1**). The reaction contained a final concentration of 1× Q5 buffer, 0.02 U/µl Q5 high-fidelity DNA polymerase (New England Biolabs Inc., Ipswich, USA), 500 nM forward and reverse primer, respectively, 100 ng of plasmid DNA, 0.2 mM dNTP mix, and ultrapure water to adjust to a final volume of 50 µl. The PCR was carried out using the program described in **Supplementary Table 1**. The amplified product was purified by agarose gel electrophoresis with subsequent gel extraction using the Monarch^®^ DNA Gel Extraction Kit (New England Biolabs Inc., Ipswich, USA) and eluting in 30 µl of ultrapure water. The concentration of the purified DNA was determined using an IMPLEN NanoPhotometer^®^ N60 (Implen GmbH, Munich, Germany). Afterwards, 10 ng of this linear DNA were used for transformation.

**Table 1:** Primers used in this study.

Primers	Sequence (5’-3’)	Tm (°C)
For synechocystis sp. PCC 6803 pirC		
1. Fw colony PCR	AGTTGCCGCATTTCCTTGAC	66
2. Rev colony PCR	CGCCGCCATCACATTGTCTC	69
3. Fw linear DNA	CAGCTATGACCATGATTACGCCAAGCTTGC ATGCCTGCAAAAATTGCGAATGCCCCGGTC	84
4. Rev linear DNA	GGCCAGTGAATTCGAGCTCGGTACCCGGGG ATCCTCTAGAACCCCGTTTCCTCCTGGATG	87
For synechococcus elongatus PCC 7942 comFB		
5. Fw colony PCR	AGCCCCTGGTCGCATCTCCA	61
6. Rev colony PCR	TGCCATGTTGCTGCGGGGTT	62
For *synechococcus* sp. UTEX 3153 *pirC*		
7. Fw *pirC* US	CGCGGGGAGATTCTAACTCAGGC	72
8. Rev *pirC* US	GCTGCGTTCGGTCAAGAGCTCGGG ATTCCTGGGTAAATATTTAAATAG	77
9. Fw *specR*	CTATTTAAATATTTACCCAGGAAT CCCGAGCTCTTGACCGAACGCAGC	77
10. Rev *specR*	GGCAAAAATTTAAGGAAAAACC ATATGGAGTTTGTAGAAACGC	71
11. Fw *pirC* DS	GCGTTTCTACAAACTCCATATG GTTTTTCCTTAAATTTTTGCC	79
12. Rev *pirC* DS	GTTCCCGGCGAAAGGTGACGAGGGG	71

Abbreviations: For, forward; Rev, reverse; US, upstream; DS, downstream.

### 2.3 Generation of linear knockout construct via overlapping extension (OE) PCR for transformation of *Synechococcus* sp. UTEX 3153

A linear *pirC* knockout cassette was constructed for *Synechococcus* sp. UTEX 3153 via overlapping extension (OE) PCR. The upstream and downstream homologous regions of the *pirC* locus of *Synechococcus* sp. UTEX 3153 and a spectinomycin resistance cassette were separately amplified with overlapping sequences using primers 7 - 12 (**Table 1**). The upstream and downstream homologous regions of *pirC* were amplified from genomic DNA extracted from *Synechococcus* sp. UTEX 3153 using a previously described method (Singh et al., 2011), and the spectinomycin resistance cassette was amplified from the pJA1 plasmid. DNA amplification and purification was carried out as described above using the PCR program shown in **Supplementary Table 2**. Afterwards, equimolar amounts (0.5 pmol) of each DNA fragment were combined in a 1.5 ml reaction tube and used as a template for OE PCR to assemble the complete *pirC* knockout cassette using the PCR program shown in **Supplementary Table 3**. A total of 10 ng of the purified linear DNA was subsequently used for BP-mediated transformation.

### 2.4 Preparation of BPP Bioportides™ with cargo DNA for transformation

The BP protein kit was purchased from the Badische Peptide und Proteine (BPP) GmbH (Mannheim, Germany) and contained a vial of lyophilized protein (1 µg) and either resuspension buffer or 50% sterile glycerol. The product was handled as recommended by the manufacturer (https://www.badischepeptide-proteine.de/technology/bioportides). To preserve the functional integrity of BP, the lyophilized protein should be stored at −80 °C to prevent hydrolysis, oxidation, and aggregation. Upon reconstitution, solutions in resuspension buffer are stable for short-term use (2–3 days). For long-term storage, proteins should be dissolved in 50% sterile glycerol and stored at −20 °C. Repeated freeze-thaw cycles should be avoided to minimize irreversible loss of activity. To prevent contamination, BP and cargo DNA were handled exclusively within a sterile laminar-flow hood. For a final BP concentration of 100 ng/µl, 10 µl of the resuspension buffer was added to the lyophilized protein aliquot. The mixture was then gently mixed by pipetting and subsequently incubated at room temperature (RT) for 20 minutes without shaking. Afterwards, 100 ng (1 µl) of this BP solution was transferred to an empty sterile 1.5 ml reaction tube, and 10 ng of linear or plasmid DNA was added. The mixture was gently mixed by pipetting and subsequently incubated at room temperature (RT) for 10 minutes without shaking. The resulting DNA-BP solution was immediately used for transformation.

### 2.5 Transformation of cyanobacterial cells using BP

50 ml of exponentially grown cyanobacterial cells with an optical density (OD) of 0.4 at 750 nm were harvested by centrifugation for 10 min at 4000 xg at room temperature. The supernatant was discarded, and the cells were resuspended in 2 ml of their respective growth medium. The transformation protocol was adapted from the one provided by the manufacturer (https://www.badischepeptide-proteine.de/technology/bioportides). For the transformation of *Synechocystis* sp. PCC 6803, a *pirC* (*sll0944*) knockout plasmid (pJA1 plasmid obtained from (Orthwein et al., 2025)) and a linear PCR product derived from that plasmid (generated as described in section 2.2) were used in combination with Bioportide 17 (BP-17) or Bioportide 12 (BP-12), respectively. From the ten different BPs that were originally designed for gene delivery in gram-negative bacteria and plants, BP12 and BP17 had previously successfully been tested in *Escherichia coli* and tobacco plants (Kutzner, 2022) and were thus selected for this study. *Synechococcus elongatus* PCC 7942 WT was transformed using a *comfB* (*Synpcc7942*−*1924*) knockout plasmid (obtained from (Samir et al., 2025)) in combination with BP-12. *Synechococcus* sp. UTEX 3153 WT was transformed using a linear *pirC*-knockout construct generated via OE PCR (section 2.3) in combination with BP-17. For all transformations, 50 µl of the prepared cyanobacterial cell suspensions were gently mixed with the appropriate BP-DNA solution by pipetting. Subsequently, the resulting mixture was incubated at RT for 10 min without shaking. After incubation, 950 µl of the respective growth medium, BG11 or AD7, was added, and the mixture was incubated horizontally under 10 µmol PAR light, shaking with 130 rpm for different time intervals. *Synechocystis* sp. PCC 6803 cells were incubated for 1 h, 6 h, and 24 h, *Synechococcus elongatus* PCC 7942 cells, and *Synechococcus* sp. UTEX 3153 was incubated for 1 h and 6 h. After incubation, the cells were spread onto an Hydrophylic Acrylic Teflon Fluorinated (HATF) membrane placed on BG11 or AD7 agar plates without antibiotics and incubated overnight under 10 µmol PAR. The membranes were transferred onto BG11 or AD7 agar plates supplemented with the appropriate antibiotics for strain selection on the following day and incubated under 20 µmol continuous PAR. Colonies of transformants appeared 7–8 days after transformation.

### 2.6 Confirmation of the transformants via colony PCR

To confirm the deletion of the respective genes (*pirC* in *Synechocystis* sp. PCC 6803 and *Synechococcus* sp. UTEX 3153, or *comfB* in *Synechococcus elongatus* PCC 7942) several randomly selected transformants from each incubation condition were analyzed via colony PCR, 7 days after transformation. Each reaction contained 5 µl Red MasterMix (2×) (Genaxxon Bioscience, Ulm, Germany), 500 nM forward and reverse primers (**Table 1**), and ultrapure water to a final volume of 10 µl. The single colonies were randomly picked with sterile micropipette tips and added to the reaction mix as templates. The reactions were carried out using a SensoQuest LabCycler™ (SensoQuest GmbH, Göttingen, Germany). For the confirmation of *pirC* deletion, the program described in **Supplementary Table 4** was used, with an annealing temperature of 67 °C and an extension time of 3 min 30 s for *Synechocystis* sp. PCC 6803 colonies and an annealing temperature of 72 °C and an extension time of 2 min 30 s for *Synechococcus* sp. UTEX 3153 colonies. To confirm the deletion of *comfB* in *Synechococcus elongatus* PCC 7942, the program listed in **Supplementary Table 5** was used. Wild-type strains were included as negative controls in each test (throughout the experiments for the confirmation) of strain segregation. The resulting PCR products were analysed by electrophoresis on a 1% agarose gel in TAE buffer containing 0.02 µl/ml Midori Green (Nippon Genetics Europe GmbH, Düren, Germany) under UV illumination.

## 3 Results

### 3.1 Mechanism of BPP-mediated transformation

**Figure 1:**
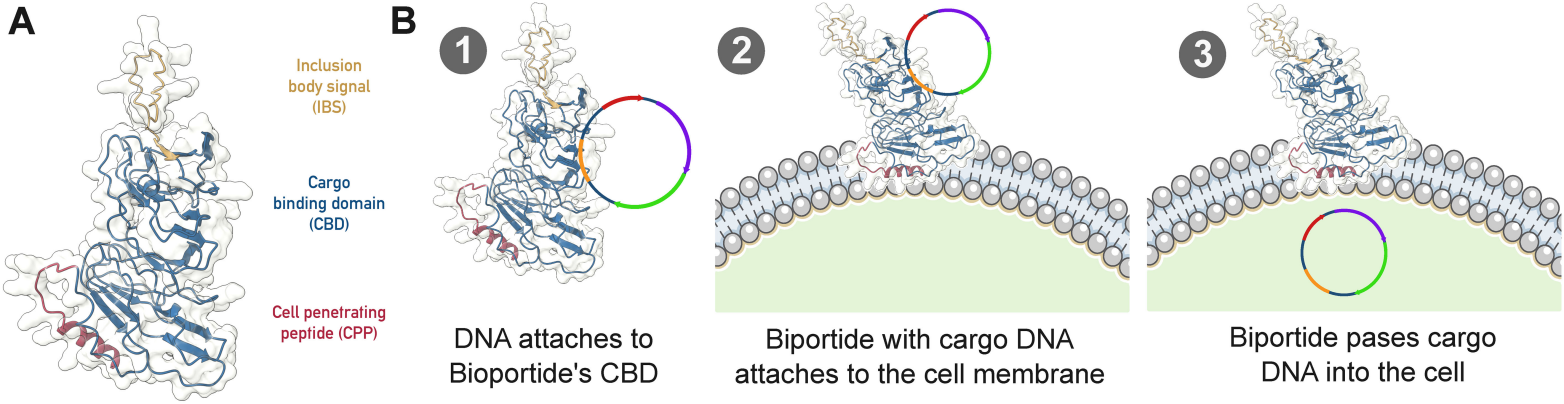
Structural and mechanistic overview of Bioportide-mediated transformation. **(A)** Predicted three-dimensional structure of BP-17, generated using AlphaFold and visualized in UCSF ChimeraX. **(B)** Schematic model illustrating the Bioportide-mediated delivery mechanism. The DNA-binding domain of BP-17 interacts with nucleic acid cargo, including linear DNA, RNA, or plasmid DNA, to form a stable complex. This complex subsequently associates with the cyanobacterial cell surface and translocates the cargo across the membrane, enabling intracellular delivery.

BPs combine two functions: i) reversible binding of various types of nucleic acids via their sugarphosphate backbone (Cargo binding domain), and ii) transport of bound nucleic acids across the cell membrane into different cell types (Cell penetrating peptide) (**Figure 1**). The different types of BPs differ in their membrane-crossing domains. These different membrane-crossing domains were explicitly designed to cross specific membrane or cell wall types, e.g., Gram-negative, Gram-positive, or strongly glycosylated (Kutzner, 2022).

The cargo binding domain of BPs exhibits a remarkable capacity to bind a wide range of nucleic acid types, including both RNA and DNA molecules (**Figure 1**). Successfully complexed nucleic acids include small interfering RNAs (siRNAs), various microRNAs, and DNA constructs such as linear integrative plasmids and circular replicative plasmids exceeding 10 kb. This broad substrate promiscuity is attributable to the unique structural properties of the BP cargo binding domain. Engineered from a polysaccharidebinding domain originally identified in an extremophilic *Clostridium* species, the domain was specifically modified for its application in nucleic acid loading within the BP framework (Kutzner, 2022).

Unlike conventional DNA- or RNA-binding domains that recognize specific nucleotide sequences, the BP does not interact with nucleobases. Instead, they bind nucleic acids by recognizing the sugar-phosphate backbone. Electrostatic interactions between the protein and the conformationally exposed sugar residues enable this association. Both ribose and deoxyribose sugars participate in these interactions via stacking and CH-*π* interactions with aromatic amino acid residues, particularly those located within the loop regions connecting the *β*-strands of the domain. Structural modelling of the BP using AlphaFold revealed a tertiary conformation resembling a broad, three-pronged fork (**Figure 1**). Each prong contains aromatic residues, most notably tryptophan, that facilitate *π*–*π* and CH–*π* interactions, creating an optimal binding interface for nucleic acid polymers (Kutzner, 2022).

Transport of the nucleic acid-BP complex into cells is facilitated by an integrated cell-penetrating domain (**Figure 1**). Upon membrane interaction, the cell-penetrating domain adopts an alpha-helical secondary structure, consistent with the behaviour of other membrane-active peptides (**Figure 1**). Although the precise mechanism of cellular entry remains to be fully elucidated, it is currently assumed that they form transient pores, using a mechanism akin to that of the membrane-active peptide melittin (Ulmschneider and Ulmschneider, 2024). In this study, two variants of BP, designated BP-12 and BP-17, were used. These variants differ in their membrane translocation sequences, each derived from well-characterized cell-penetrating peptides previously developed and validated by BPP GmbH. These BPs have demonstrated efficient cellular uptake and functional delivery in both plant and bacterial systems (Kutzner, 2022).

### 3.2 Optimization of BP-mediated transformation of *Synechocystis* sp. PCC 6803 and *Synechococcus elongatus* PCC 7942 using plasmid DNA

**Figure 2:**
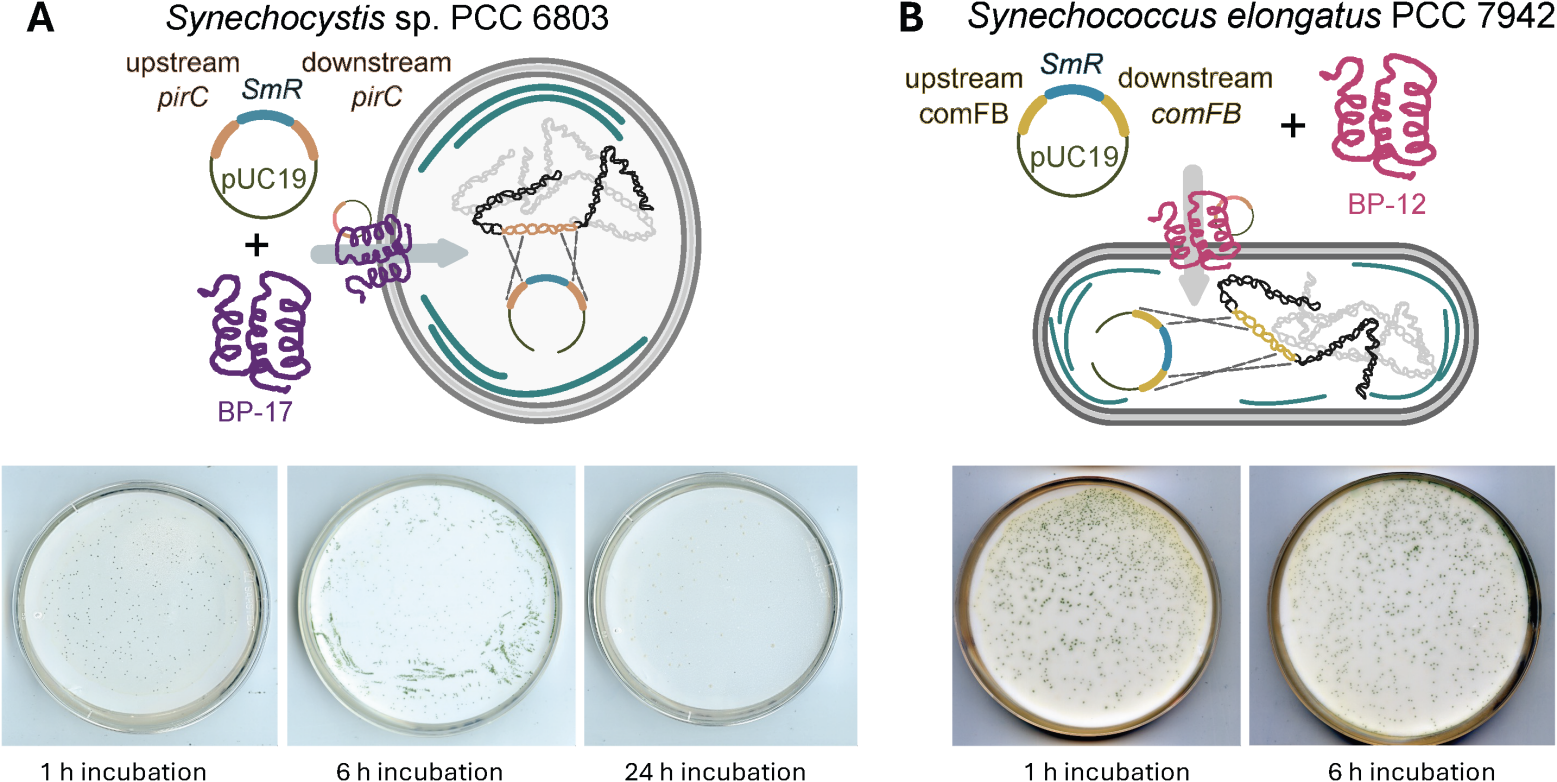
Bioportide-mediated transformation of *Synechocystis* sp. PCC 6803 and *Synechococcus elongatus* PCC 7942. **(A)** Transformation of *Synechocystis* with BP-17 for *pirC* knockout. Cells were incubated with 100 ng of BP and 10 ng of plasmid for three different time intervals (1 h, 6 h, and 24 h) before plating on BG11 agar medium without antibiotics. After 24 h of incubation, the membranes were transferred on BG11 agar medium supplemented with 50 µg/ml spectinomycin for selection. **(B)** Transformation of *S. elongatus* with BP-12 for *comFB* knockout. Cells were incubated with 100 ng of BP and 10 ng of plasmid for two different time intervals (1 h and 6 h) before plating on BG11 agar medium without antibiotics. After 24 h of incubation, the membranes were transferred on BG11 agar medium supplemented with 50 µg/ml spectinomycin for selection.

We first aimed to establish working protocols for efficient BP-mediated transformation of *Synechocystis* sp. PCC 6803 and *Synechococcus elongatus* PCC 7942, both widely used model cyanobacterial strains, using low plasmid DNA input. Bioportide 17 (BP-17) was used for transformation of *Synechocystis*, along with 10 ng of plasmid DNA (pJA1 (Orthwein et al., 2025)), to generate a *pirC* knockout strain by replacing the *pirC* gene with a spectinomycin-resistance cassette. PirC, a regulatory protein involved in controlling carbon partitioning in cyanobacteria, was selected as a model target in this study since it represents an interesting target for metabolic engineering. In addition, *pirC* is non-essential, and deletion strains do not show severe vegetative phenotypes, making it suitable for testing transformation efficiency (Orthwein et al., 2025). For *S. elongatus*, Bioportide 12 (BP-12) and 10 ng of plasmid DNA (pUC19Δ7942*comFB* (Samir et al., 2025)) were used for transformation to create a *comFB* knockout strain by replacing the *comFB* gene with a spectinomycin-resistance cassette. *ComFB* is a signaling protein required for pilus biogenesis and DNA uptake (Samir et al., 2025). It was selected as a model target because, like PirC, it is non-essential. The negative controls included WT cells without BP-17/BP-12 or plasmid DNA, WT cells with 10 ng of plasmid DNA without BP-17/BP-12, and WT cells with BP-17/BP-12 without plasmid DNA (**Supplementary Figure 1**). No transformant colonies were observed under these conditions, indicating that none of the strains can be naturally transformed with only 10 ng of plasmid DNA. Furthermore, the absence of transformants after incubation of WT cells with BP-17/BP-12 in the absence of plasmid DNA shows that BP does not confer antibiotic resistance. Since previous publications had shown that incubation time strongly influences transformation efficiency (Zang et al., 2007), we tested BP-mediated transformation by incubating *Synechocystis* WT cells with BP-17 and plasmid DNA for 1 h, 6 h, and 24 h under constant low light at 30 °C with shaking at 120 rpm. The transformation efficiency increased from 1 h to 6 h, but significantly decreased after 24 h of incubation (**Table 2** and **Figure 2**). Given these results, incubation for 24 h was not applied for future experiments. For *S. elongatus*, WT cells were incubated with BP-12 for 1 h and 6 h under constant low light at 30 °C with shaking at 120 rpm. As for *Synechocystis*, the transformation efficiency also increased from 1 h to 6 h of incubation (**Table 2** and **Figure 2**).

**Table 2:** Colony counts following Bioportide transformation with various incubation times and different DNA types as cargo.

Organism	Type of DNA	Incubation time	No. of colonies
*Synechocystis* sp. PCC 6803	Plasmid	1h	199
		6 h	351
		24 h	102
*Synechococcus elongatus* PCC 7942	Plasmid	1h	325
		6 h	473
*Synechocystis* sp. PCC 6803	Linear	1h	3
		6 h	2
*Synechococcus* sp. UTEX 3153	Linear	1h	89
		6h	36

**Figure 3:**
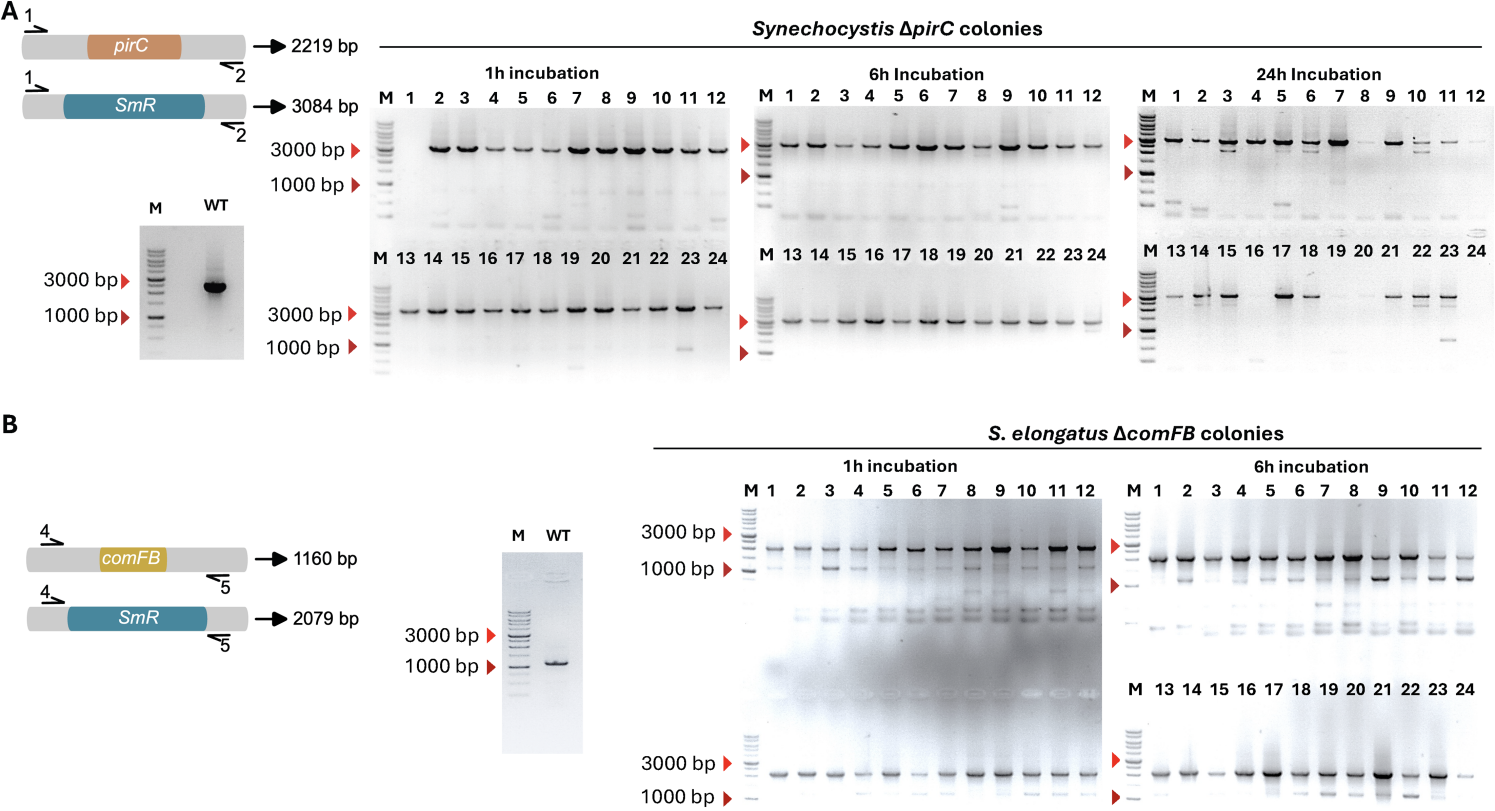
Confirmation of gene insertion in transformed cells via PCR. **(A)** Agarose gels of the products of colony PCRs of the WT and 24 randomly selected transformants from each incubation condition confirmed the knockout of the *pirC* gene in *Synechocystis* sp. PCC 6803. **(B)** Agarose gels of the products of colony PCRs of the WT and 24 randomly selected transformants from each incubation condition confirmed the knockout of the *comfB* gene in *Synechococcus elongatus* PCC 7942. Colony PCR was performed seven days post-transformation.

To confirm the integration of exogenous DNA at the target site (*pirC* locus for *Synechocystis* and *comFB* locus for *S. elongatus*) in the host genome by double homologous recombination, 24 randomly selected independent spectinomycin-resistant (Spec*^R^*) transformants from each of the three incubation times were chosen. After selection through two rounds of selective culturing on BG11 agar plates supplemented with spectinomycin, they were analysed via colony PCR followed by agarose gel electrophoresis (**Figure 3**). For *Synechocystis* WT cells, a 2219-bp DNA fragment was expected to be amplified from the *pirC* locus, whereas for Spec*^R^* transformants with the *pirC::specR* genotype, a 3084-bp fragment was expected (**Figure 3**). Colony PCR analysis confirmed the presence of a 3-kb product in all transformants examined from the 1 h and 6 h incubation transformation plates, while a 2.5-kb product was observed for the WT control. This indicated that in all Spec*^R^* transformants obtained from the 1 h and 6 h incubation, the *specR* gene was stably integrated into the *pirC* locus, and two rounds of selection in BG11 with spectinomycin resulted in complete segregation of the transformed genome. However, in transformants from the 24 h incubation, both completely and partially segregated transformed genomes were observed. For *S. elongatus* WT cells, a 1160-bp DNA fragment was expected to be amplified from the *comfB* locus, whereas for Spec*^R^* transformants with the *comfB::specR* genotype, a 2079-bp fragment was expected. Colony PCR analysis confirmed the presence of a 2-kb product in all examined transformants, while a 1.2-kb product was observed in the WT control and some of the transformants (**Figure 3**). These results indicate that in all Spec*^R^* transformants obtained from the 1 h and 6 h incubation, the *specR* gene was stably integrated into the *comFB* locus. However, two rounds of selection in BG11 with spectinomycin yielded both completely and partially segregated transformed genomes. These findings indicate that BP enables the efficient transformation of *Synechocystis* and *S. elongatus*, even at a low plasmid DNA concentration of approximately 10 ng/ml and a short incubation time of 1 h.

### 3.3 BP-mediated transformation of *Synechocystis* sp. PCC 6803 using linear DNA

**Figure 4:**
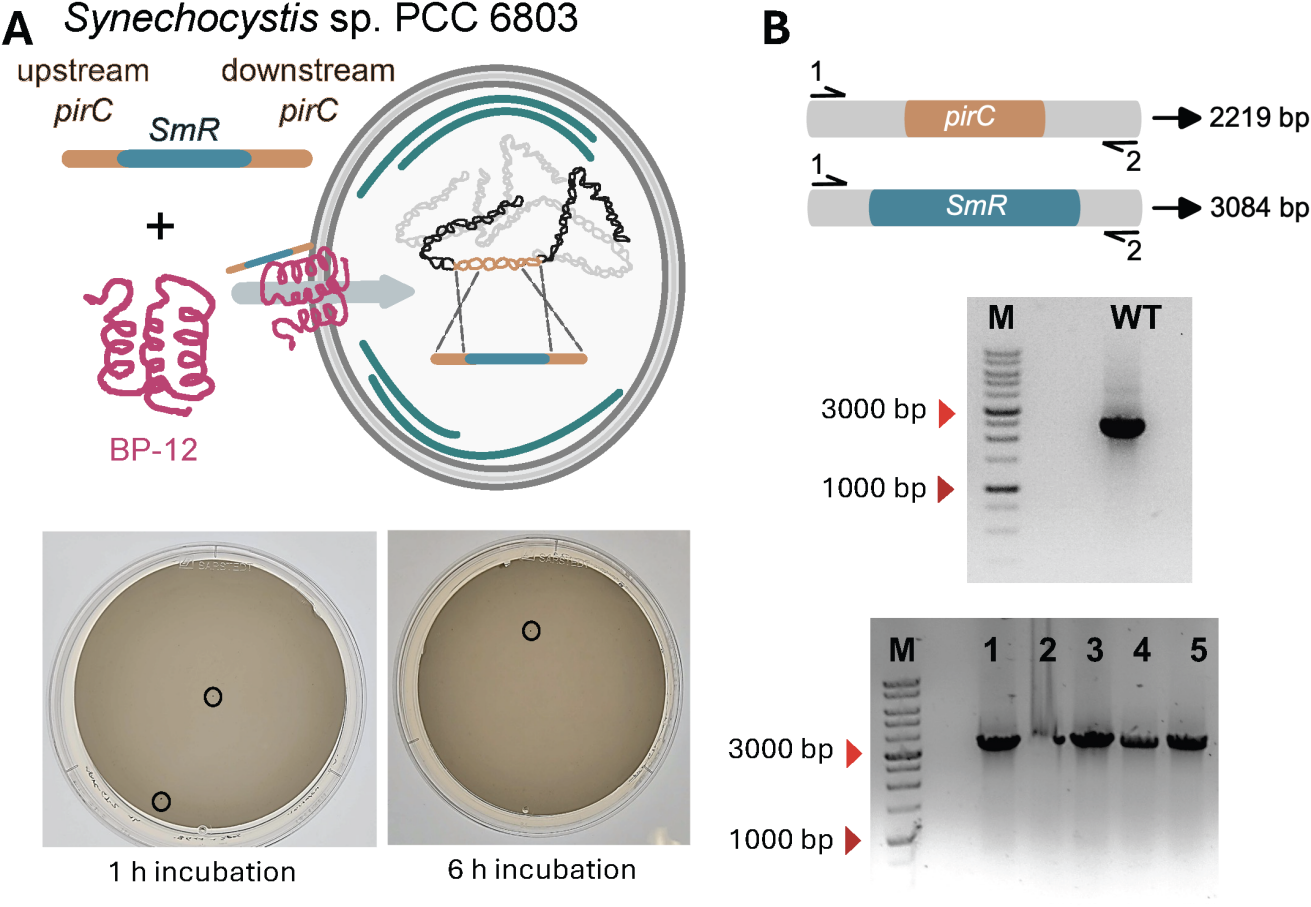
Bioportide-mediated transformation of a linear PCR product in *Synechocystis* sp. PCC 6803. **(A)** Cells were incubated with 10 ng of linear DNA and 100 ng of Bioportide (BP) for 1 h and 6 h and plated on BG11 agar medium without antibiotics. After 24h incubation, cells were transferred to BG11 agar plates supplemented with 50 µg/ml spectinomycin for selection. Colonies are marked with circles. **(B)** Colony PCR analysis of five randomly-selected transformants performed seven days post-transformation.

Previous studies have shown that linear DNA fragments can be used effectively for genetic modification in *Synechocystis* (Nagarajan et al., 2011; Kufryk et al., 2002). Transformation using linear PCR products offers several advantages, including a streamlined workflow, reduced time and cost requirements, and the elimination of cloning steps. To test BP-mediated transformation of linear PCR products, the *pirC* knockout cassette was amplified from the pJA1 plasmid (**Figure 4**). *Synechocystis* cells were incubated with BP-12 and 10 ng of the amplified linear PCR product for 1 h and 6 h, leading to successful transformation in both conditions (**Figure 4**). Negative controls included WT cells alone, WT cells with 10 ng of linear PCR product in the absence of BP-12, and WT cells with BP-12 but without the linear PCR product (**Supplementary Figure 2A**). No transformant colonies were observed under these conditions. Interestingly, the transformation efficiency for *Synechocystis* was considerably lower when using a linear PCR product than when using plasmid DNA (**Table 2**).

To confirm the integration of exogenous DNA at the *pirC* locus in the host genome, all independent Spec*^R^* transformants were selected through two rounds of selective culturing on BG11 agar plates supplemented with spectinomycin and analysed by colony PCR followed by agarose gel electrophoresis as described above. A 3-kb product was detected for all transformants, while a 2.5-kb product was observed for WT cells, indicating stable integration of *specR* into the *pirC* locus and full segregation of the transformants (**Figure 4**).

### 3.4 BP-mediated transformation of *Synechococcus* sp. UTEX 3153 using a linear PCR product generated by OE PCR

**Figure 5:**
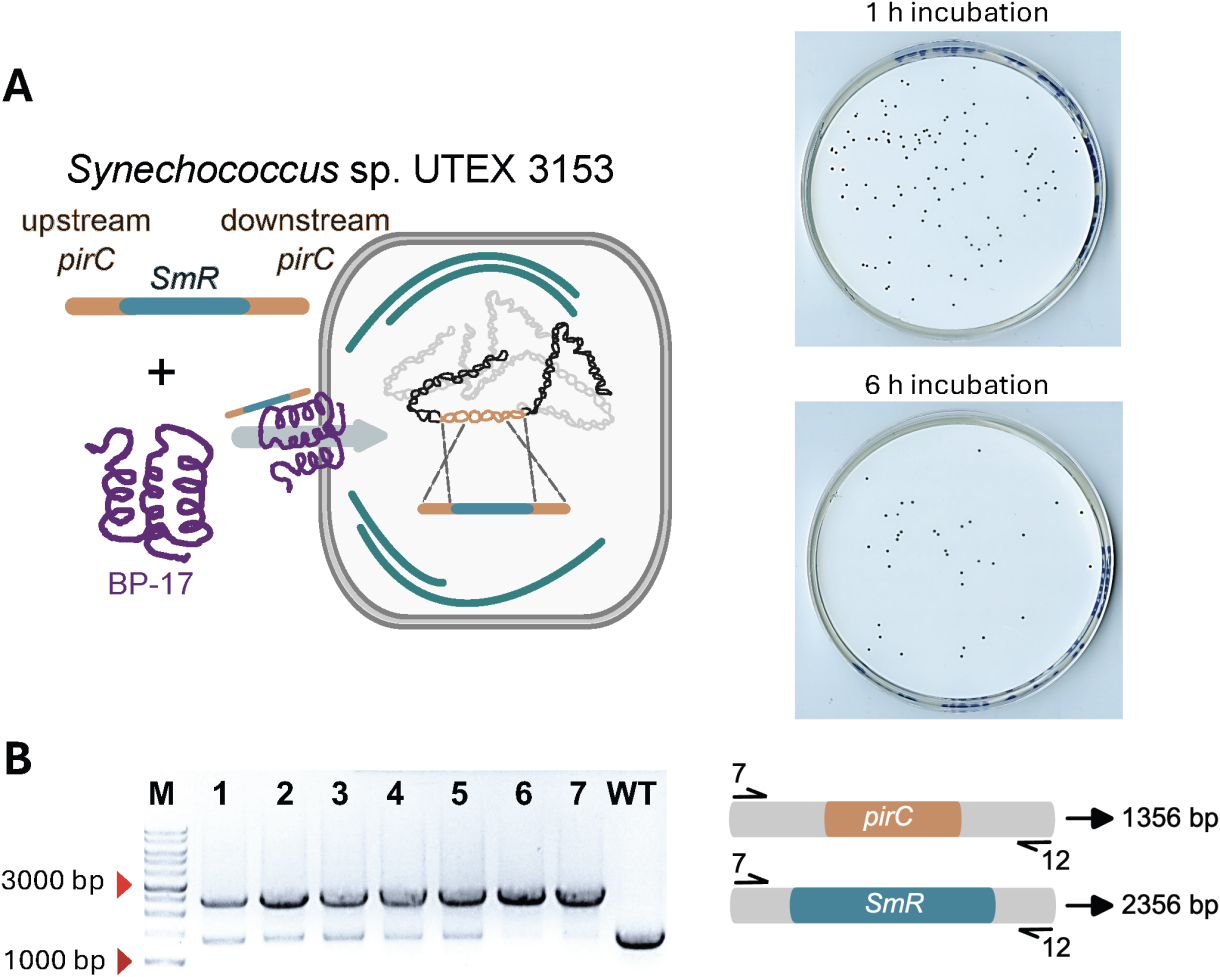
Bioportide-mediated transformation of a linear PCR product in *Synechococcus* sp. UTEX 3153. **(A)** Cells were incubated with 10 ng of linear DNA and 100 ng of Bioportide (BP) for 1 h and 6 h and plated on AD7 agar medium without antibiotics. After incubation for 1 h and 6 h, the cells were plated on AD7 agar medium supplemented with 50 µg/ml spectinomycin. **(B)** Colony PCR analysis of seven randomly selected transformants performed seven days post-transformation.

The strain *Synechococcus* sp. UTEX 3153 is a fast-growing marine cyanobacterium with industrial potential for which no transformation protocol has been established so far (Włodarczyk et al., 2020). The results obtained for *Synechocystis* demonstrated that linear DNA fragments can be introduced in the cell via BP-mediated transformation. Building on this approach, we sought to establish a BP-mediated transformation method for *Synechococcus* sp. UTEX 3153 using linear DNA. OE PCR was used to construct a linear knockout cassette targeting the *pirC* gene in *Synechococcus* sp. UTEX 3153. Transformants were obtained after incubation of cells with 10 ng of this linear construct along with 100 ng of BP-17 for 1 h and 6 h (**Figure 5**). These results confirm that BP-17 also facilitates the transformation of WT *Synechococcus* sp. UTEX 3153 cells using linear PCR products, even at a low DNA concentration. The negative controls included WT cells alone, WT cells with 10 ng of linear PCR product in the absence of BP-17, and WT cells with BP-17 but without the linear PCR product. No colonies were observed under these conditions (**Supplementary Figure 2B**).

To confirm the integration of exogenous DNA at the *pirC* locus in the host genome, seven independent Spec*^R^* transformants were selected through two rounds of selective culturing on AD7 plates supplemented with 50 µg/mL spectinomycin and afterward analysed by colony PCR and agarose gel electrophoresis (**Figure 5**). For WT cells, a 1356 bp DNA fragment was expected to be amplified from the *pirC* locus, while for Spec*^R^* transformants with the *pirC::specR* genotype, a 2356-bp fragment was expected. Colony PCR analysis confirmed the presence of a 2.5 kb product for all examined transformants, while a 1.5 kb product was observed for the WT control. These results confirm that in all Spec*^R^* transformants obtained from the 1 h and 6 h incubation, the *specR* gene was stably integrated into the *pirC* locus. However, for some transformants, the 1.5 kb WT product was additionally observed, suggesting that two rounds of selection in AD7 with spectinomycin resulted in both completely and partially segregated transformed genomes.

## 4 Discussion

Despite considerable advances in cyanobacterial genetic engineering, several inherent and methodological limitations continue to hamper efficient and reproducible genome modification across different strains. This study provides a systematic evaluation of BP-mediated transformation across multiple cyanobacterial strains, including *Synechocystis* sp. PCC 6803, *Synechococcus elongatus* PCC 7942, and *Synechococcus* sp. UTEX 3153. Our findings highlight the utility and versatility of BP in facilitating genetic manipulation in cyanobacteria, which have traditionally presented challenges in transformation efficiency, particularly when low DNA concentrations or linear DNA fragments are used.

*Synechocystis* sp. PCC 6803 is widely used as a model cyanobacterium in both fundamental and applied research because of its natural competence, ability to grow heterotrophically on glucose, and the availability of the fully sequenced and annotated genome (Doello et al., 2026). *Synechococcus elongatus* PCC 7942 is the most extensively studied prokaryotic model organism for circadian rhythms (Cohen and Golden, 2015; Taton et al., 2020) and was the first cyanobacterial strain to be reliably transformed by exogenously added DNA (Van Den Hondel et al., 1980). Exogenous DNA introduced into *Synechocystis* and *S. elongatus* via natural transformation integrates into the genome through double homologous recombination (Grigorieva and Shestakov, 1982), making them valuable organisms in biotechnology, where engineered strains are used to produce various industrially relevant chemicals (Ducat et al., 2011 Doello et al., 2026; Żymańczyk Duda et al., 2022). However, established protocols for natural transformation of these strains are time-consuming and require high concentrations of exogenous DNA for effective transformation (Zang et al., 2007).

Optimization of transformation conditions for *Synechocystis* and *S. elongatus* using BP revealed several key insights. First, the absence of transformant colonies in control conditions confirms that these strains, while naturally competent under specific conditions, are not amenable to transformation with only 10 ng of plasmid DNA in the absence of BP. These findings align with prior observations that transformation efficiency in cyanobacteria is highly dependent on DNA concentration, DNA delivery mechanisms, and the physiological state of the cells (Zang et al., 2007). The observed increase in transformation efficiency with incubation time from 1 h to 6 h, followed by a decrease at 24 h, suggests a time-sensitive window for optimal DNA uptake. This is consistent with previous studies indicating that prolonged exposure to transforming agents can lead to cellular stress or DNA degradation, thereby reducing efficiency (Zang et al., 2007).

Cyanobacteria are polyploid organisms, harbouring multiple copies of their chromosome (Schirrmeister et al., 2012). Initial recombination events may only integrate the foreign DNA into a subset of genome copies, leading to a heterogeneous population with both WT and transformed alleles (partial seggregation). In our experiments, colony PCR confirmed successful integration of the *specR* construct into the corresponding loci, with full segregation achieved after two rounds of antibiotic selection in most of the 1 h and 6 h transformants for *Synechocystis*. However, for *S. elongatus*, as well as for the 24 h incubated *Synechocystis* cells, some transformants displayed partial segregation. These observations highlight that while BP-enhanced delivery is effective, post-transformation culturing conditions remain a critical factor influencing genome stability and editing efficiency in cyanobacteria. To mitigate this, serial subculturing under increasing antibiotic pressure could be used to gradually enrich fully segregated transformants. Prolonged incubation under selective pressure, ideally under conditions that support slower growth and fewer genome copies (e.g., low light or phosphate limitation), can also aid in achieving full replacement of wild-type loci (Pope et al., 2020).

While BP effectively mediates intracellular delivery of plasmid DNA into cyanobacterial cells, it does not protect against degradation by host-encoded R-M systems (Porter, 1986). Cyanobacteria possess native restriction endonucleases that selectively recognize and cleave unmethylated or foreign DNA at specific recognition sequences, thereby posing a significant barrier to successful transformation when such motifs are present within the plasmid construct (Hren et al., 2025). The abundance and types of endogenous R-M systems differs across cyanobacterial strains (Zhao et al., 2006; Papoulis et al., 2021), which can lead to different segregation efficiency after successful DNA uptake. Therefore, as with other transformation methods, conducting *in silico* analysis of plasmid sequences before BP-mediated transformation is vital to identify and eliminate internal restriction sites that correspond to known host enzymes. This can be achieved through site-directed mutagenesis strategies or by cloning the plasmid into a methylation-competent *Escherichia coli* strain, which can pre-modify the plasmid DNA at recognition sites to prevent cleavage.

Since time is a major constraint in the genetic engineering of cyanobacteria, the use of linear DNA fragments amplified by PCR would offer a streamlined, cloning-free, and therefore faster method for genome editing (Almeida et al., 2017). We could show that, although with a lower efficiency than plasmid DNA, BP-mediated delivery enables the uptake of linear DNA by *Synechocystis*, even at low concentrations. The pattern of transformation efficiency between 1 h and 6 h incubation followed a similar trend for linear and plasmid DNA, suggesting that cellular uptake kinetics rather than DNA structure may be the dominant factor influenced by incubation duration under these conditions.

The marine cyanobacterial strain *Synechococcus* sp. UTEX 3153 (also known as *Synechococcus* sp. PCC 11901), a recently isolated organism, has been optimized for high-density cultivation. It exhibits rapid specific growth rates, elevated biomass productivity, and resilience to various abiotic stressors, including high light intensity and salinity. These physiological characteristics make it a promising candidate for largescale biotechnological applications, particularly in outdoor, non-axenic cultivation systems (Włodarczyk et al., 2020). Despite its potential, the development of robust genetic tools for this strain remains limited. We could successfully conduct BP-mediated transformation of linear DNA into *Synechococcus* sp. UTEX 3153, demonstrating the feasibility of extending BP-mediated delivery to non-model but industrially relevant strains.

In this study, the variants BP-17 and BP-12 showed effective delivery of plasmid DNA into *Synechocystis* sp. PCC 6803, *Synechococcus elongatus* PCC 7942, and *Synechococcus* sp. UTEX 3153, potentially due to favourable interactions with the anionic, multilayered structure of the cyanobacterial cell envelope (Hoiczyk and Hansel, 2000). However, the efficiency of other BP variants may vary with parameters such as membrane permeability, surface electrostatics, and cargo properties, necessitating empirical validation for new host strains or genetic constructs.

Overall, BP-mediated transformation enables faster and more efficient cyanobacterial transformation while using only a fraction of the DNA necessary for natural transformation. Although electroporation can be performed with similar or even lower DNA amounts of 0.1–16 µg (Ludwig et al., 2008), BP-mediated transformation does not require time-consuming washing steps to prepare cells, nor does it need specialized electroporation equipment. While the material cost of approximately 20 € per transformation is higher than for natural transformation, the BP workflow reduces cloning steps, DNA concentration and incubation time, compensating for the reagent cost. Consequently, BP-mediated transformation represents a compelling alternative to conventional methods such as electroporation and chemical transformation, particularly for recalcitrant strains. As such, BPP Bioportides™ significantly expand the molecular toolkit available for cyanobacterial synthetic biology and biotechnology.

## Data Availability

The original contributions presented in the study are included in the article/**Supplementary Material**. Further inquiries can be directed to the corresponding author.
