# Supplementary material for: BPP Bioportide™-mediated (genetic) transformation in cyanobacteria: a rapid and simplified approach for efficient molecular translocation and genome modification
Source: Front Plant Sci. 2026 Apr 22;17:1812316. doi: 10.3389/fpls.2026.1812316 (PMC13147198; doi:10.3389/fpls.2026.1812316)
Supplement: Supplementary file 1 [file DataSheet1.pdf]

Supplementary Material

1 SUPPLEMENTARY TABLES AND FIGURES

Step	Temperature (°C)	Time (s)	Cycles
Initial denaturation	95	60	1
Denaturation	95	30	35
Annealing	67	30	35
Extension	72	103	35
Final extension	72	600	1

Table S1. PCR program using Q5 high-fidelity DNA polymerase to generate one linear PCR product

Step	Temperature (°C)	Time (s)	Cycles
Initial denaturation	95	120	1
Denaturation	95	30	30
Annealing	67	30	30
Extension	72	20-30 s/kb	30
Final extension	72	600	1

Table S2. PCR program using Q5 high-fidelity DNA polymerase to generate the DNA fragments for the overlapping extension PCR

Step	Temperature (°C)	Time (s)	Cycles
Initial denaturation	95	120	1
Denaturation	95	30	35
Annealing	67	30	35
Extension	72	150	35
Final extension	72	600	1

Table S3. PCR program using Q5 high-fidelity DNA polymerase overlapping extension PCR

Step	Temperature (°C)	Time (s)	Cycles
Initial denaturation	95	900	1
Denaturation	95	30	30
Annealing	53-72	30	30
Extension	72	20-30 s/kb	30
Final extension	72	600	1

Table S4. Colony PCR program for the confirmation of *pirC* deletion

Step	Temperature (°C)	Time (s)	Cycles
Initial denaturation	95	900	1
Denaturation	95	30	30
Annealing	55	30	30
Extension	72	120	30
Final extension	72	600	1

Table S5. Colony PCR program for the confirmation of *comFB* deletion

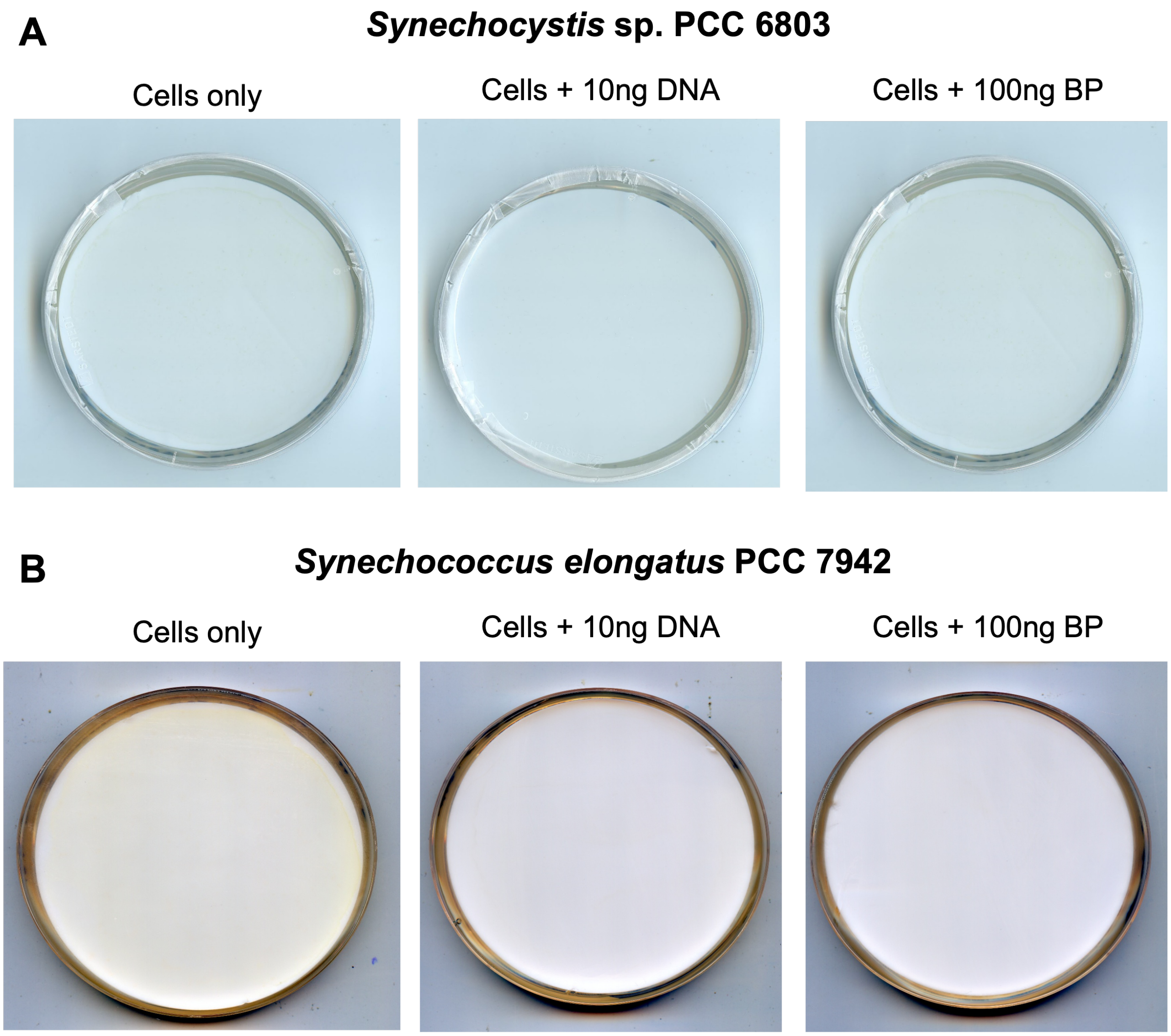

Figure S1. Negative controls for Bioportide (BP)-mediated transformation of *Synechocystis* sp. PCC 6803 (A) and *Synechococcus elongatus* PCC 7942 (B) using plasmid DNA. For both strains, WT cells, WT cells supplemented with 10 ng plasmid DNA alone, and WT cells treated with 100 ng BP alone were included as negative controls. Following 24 h incubation on antibiotic-free BG11 agar, membranes were transferred to BG11 agar supplemented with 50 μg/mL spectinomycin for selection.

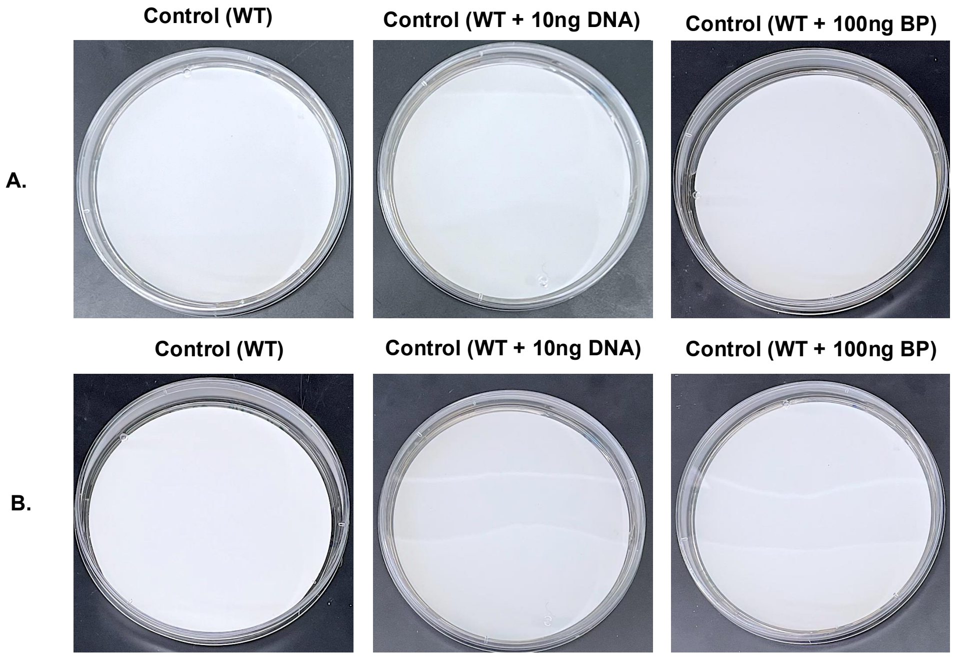

Figure S2. Negative controls for Bioportide (BP)-mediated transformation of *Synechocystis* sp. PCC 6803 (A) and *Synechococcus* sp. UTEX 3153 (B) for *pirC* gene deletion using linear DNA. For both strains, WT cells, WT cells supplemented with 10 ng linear DNA alone, and WT cells treated with 100 ng BP alone were included as negative controls. Following 24 h incubation on antibiotic-free BG11 or AD7 agar, membranes were transferred to BG11 or AD7 agar supplemented with 50 µg/mL spectinomycin for selection.
